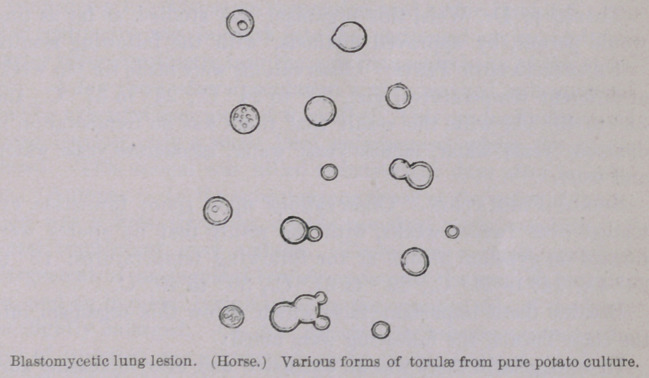# A Tumor-like Lesion in the Lung of a Horse Caused by a Blastomyces (Torula)*This article could not be completed in time to appear in “The Second Annual Report of the Cancer Committee to the Surgical Department of the Harvard Medical School.” Read, March 29, 1902, at the Second Annual Meeting of the American Association of Pathologists and Bacteriologists, at Cleveland, Ohio.

**Published:** 1902-12

**Authors:** Langdon Frothingham

**Affiliations:** Austin Teaching Fellow in Bacteriology in Harvard University


### A TUMOR-LIKE LESION IN THE LUNG OF A HORSE CAUSED BY A BLASTOMYCES (TORULA).*

By Langdon Frothingham, M.D.V.,
AUSTIN TEACHING FELLOW IN BACTERIOLOGY IN HARVARD UNIVERSITY.

(From the Bacteriological Laboratory, Harvard Medical School.)

The literature of blastomycetic infection has been so thoroughly quoted by recent writers, especially Ricketts[1] and Nichols,[2] that I refer those interested to these authors, and will mention only two works here, since they deal especially with blastomycetic lesions in animals.

Fermi and Aruch[3] describe a pseudoglanders in the horse known as *Linfangite epizootica*, *Farcin de rivière*, or *Farcin d'Afrique*. The disease closely resembles glanders and farcy, with the exception that the lungs are practically never involved. From the lesions a blastomyces was obtained which grew readily upon potato, the colonies at the end of three days being elevated and dirty white, with a smooth, non-glistening surface. On agar, gelatin, etc., there was an exceedingly scanty growth. Horses, rabbits, and guinea-pigs were inoculated with this organism, with negative results, unless we except rabbits, which were inoculated

* This article could not be completed in time to appear in "The Second Annual Report of the Cancer Committee to the Surgical Department of the Harvard Medical School." Read, March 29, 1902, at the Second Annual Meeting of the American Association of Pathologists and Bacteriologists, at Cleveland, Ohio.

in the testicle with pure cultures, after they had received an injection of ten cubic centimetres of 1 per cent. lactic acid and 30 per cent. grape sugar, three times a week for three weeks; after inoculation they again received injections of the above solution for two weeks longer, and showed suppuration in the testicle and between the abdominal muscles. An unsatisfactory illustration of the specific organism leaves its classification in doubt.

Tokishige[4] describes a pseudoglanders occurring in horses and cattle in Japan, which, he says, is identical with *Lymphangitis epizootica*. The skin lesions are the more common, and are sharply defined nodules from a pea to a walnut in size, which may remain hard, but usually suppurate and ulcerate. The lymph nodes near infected regions are generally involved. There seems to be a predilection for the testicle, where the process begins on the scrotum and prepuce, extending thence to the organ itself. The focus in the testicle is defined and resembles a tumor. Glanders-like lesions are often seen upon the nasal mucous membrane, and may extend from here to the pharynx, larynx, and trachea; they are seldom seen in the larger bronchi and never in the small, yet the lungs may rarely be involved. (The lung lesions are not described.) In all diseased parts Tokishige found blastomycetes either free or in leucocytes (from his plates I should question the character of the including cells). He obtained pure cultures of this organism upon different media. The growth upon agar was hardly visible before thirty days, and after forty to fifty days the colonies were one to four millimetres in diameter, white and compact; it was difficult to remove a small portion with the needle and to crush it under the cover-glass. In gelatin, colonies appeared after fifty-six days. Upon potato the growth was more rapid and of a light brown color (the time is not mentioned). Tokishige made many inoculation experiments, using a number of guinea-pigs, rabbits, dogs, cats, calves, horses, and swine. Pure cultures were used in some of these experiments, and in others direct inoculation from diseased portions, such as the contents of ulcers, nasal secretions, and portions of nodules from the testicle, was attempted. Inoculations were made in a variety of ways and feeding experiments were included, but the results were negative. His illustrations show that the organism which he isolated forms a mycelium.

Gross Lesion. The lesion which prompts this communication and the organism causing it to differ essentially from those just described, and, as far as I have examined the literature, nothing analogous has been recorded. This supposed tumor was situated in the posterior portion of the caudal lobe of the right lung of a horse, and was about as large again as the human head. A cut made through this large nodule showed its central portion to consist of a fairly firm gelatinous mass of a delicate pinkish-yellow color in its living portions, and containing large and small areas of necrosis. It so closely resembled a myxosarcoma that several pathologists unhesitatingly made this macroscopic diagnosis. This central portion was here and there loosely attached to a dense connective tissue wall, in other places separated from it—sequestrum-like—by a thick, yellowish, and sometimes reddish mucoid material not unlike sputum. Removal of the central mass left a cavity, to the wall of which still adhered portions of the central part of the growth. This wall consisted of dense connective tissue varying in thickness from six to less than one centimetre. Posteriorily this thick capsule gradually gave way to more or less normal lung tissue, in which small white foci were distinctly visible, and which resembled miliary tubercles or areas of chronic bronchopneumonia. (Microscopic examination of these foci gave the typical picture of chronic bronchopneumonia ; no blastomycetes were associated with the process.) In the thick wall of the lesion were a number of openings, the largest about four centimetres in diameter, which contained more or less of the sputum-like material above referred to. These communicated with each other and probably with the lung beyond, and it is assumed that they were dilated bronchi, though the specimen was so mutilated when it reached me that its true relationships could not be studied That they undoubtedly were connected with the bronchi is borne out by the clinical history of the animal ; for a year before he was killed an ever-increasing discharge from the nostrils was observed, particularly diffuse during exercise. For this reason he was suspected of having glanders, and twice, a few months previous to his death, guinea-pigs were inoculated in the usual manner for diagnosing glanders, the swab having been taken from this horse's nose. Both tests resulted in the death of the guinea-pigs from peritonitis. Whether there were metastases in the kidneys or other organs of this horse is unknown, as only the lungs were examined by the person making the autopsy.

Histology. A microscopic examination of the central or myxomatous-like portion of the lesion showed it to consist of delicate trabeculæ of connective tissue which formed a network, in the meshes of which was a varying number of cells, some probably endothelial, others connective tissue cells, and a great number of blastomycetes. In some parts the cells were quite numerous, in others almost entirely lacking, and here also the connective tissue threads were scarce, leaving a picture of quantities of blastomycetes embedded in a homogeneous, gelatinous mass. Such a mass always surrounds the blastomycetes; sometimes it is not plentiful and difficult to perceive, again particularly prominent and often wrinkled or puckered, perhaps due to contraction caused by the hardening reagents. In the more cellular parts of the lesion numerous giant cells were present. These cells varied much in size, and were often phagocytic, sometimes containing several blastomycetes. The blastomycetes varied much in size and were surrounded by a definite membrane, often showing a double contour. Within some of the blastomycetes numerous small granules of fat were often seen; others contained only one larger or smaller granule. In numerous sections made through different portions of the lesions, the above histological appearances were constant.

Blastomycetic lung lesion. (Horse.) Various forms of torulæ from pure potato culture.

CULTURES. From the lesion, pure cultures of a blastomyces were obtained upon potato, and from this transfers were made to a variety of media. Gelatin plates showed white, pin-head colonies in five to seven days, which never increased much in size as time elapsed. The surface colonies were distinctly elevated. The growth on serum and agar is white and quite marked after forty-eight hours at 37° C., but ceases to develop very much thereafter, whether kept at 37° C. or at the room temperature. The best development occurs upon potato at 25° C., and the growth continues at this temperature or at that of the room, only limited in extent by the general laws surrounding the increase of organisms in cultures. On this medium the growth is at first white, soon becoming a dirty gray, and after a few days gradually taking on a chocolate-brown color. In old cultures the growth upon the less nutritive portions of the medium becomes white and dry and resembles lime deposits. The color varies quite widely, sometimes remaining a lighter or darker yellow, again assuming the deep brown color almost at once. Such variations are the rule with many forms of torulæ.

A MICROSCOPIC EXAMINATION OF PURE CULTURES shows round organisms sometimes slightly oval which vary very much in size. The young forms are surrounded by a delicate membrane which in older forms become much thicker. The blastomycetes are sometimes surrounded by a gelatinous covering (network[5]). Fat granules are common within the cells, one or two drops may be present, or numerous small granules. Vacuoles are also observed.

Thanks to Dr. Weis, this organism was studied, as far as time would permit, by approved methods,[6] with the following results:

FERMENTATION TESTS. Observations were made for ten weeks at different temperatures, viz., 20° to 24° C., 24° to 37° C., and 38° to 40° C., with the result that there was no fermentation in dextrose yeast water, lactose yeast water, saccharose yeast water, and wort.

SPORE FORMATION. Observations upon spore formation extended over twelve weeks, with the result that no spores were formed on gypsum blocks at the following temperatures: 19° to 24° C., 24° to 27° C., 29° to 37° C., 30° to 40° C.

Besides these important facts which throw this organism into the class Torula, the following were noted:

In wort gelatin there was an extensive and ever-increasing surface growth which sank with the gradual liquefaction of the medium.

Wort is the best medium for growth.

The organism is facultative anaërobic.

No mycelia were observed.

There were many "resting cells."

This torula resembles in many ways the two torulæ of Sanfelice and those of Klein and Plimmer, which have been so carefully compared by Weis.[5] Whether an identity can be established between this organism and the four just mentioned, further study must determine.

Inoculations. A few rabbits and guinea-pigs were inoculated, some by the direct method with material obtained from the lesion in the horse, others with pure cultures of the torula.

### INOCULATIONS BY THE DIRECT METHOD.

*Rabbit* 1. A bit of the myxosarcomatous portion of the lesion was ground up with sterile water, and the suspension thus obtained was injected into the rabbit, one-half cubic centimetre being placed beneath the skin of the abdomen and one cubic centimetre introduced into the ear vein. The animal died fourteen days later. Autopsy. There was no subcutaneous lesion. Both lungs were affected with pneumonia, with some abscess formation. Smears from the thick, white contents of the bronchi showed no blastomycetes. The other organs were normal. A microscopic examination of the lungs showed a more or less chronic pneumonia and a few scattered blastomycetes. It is probable that these organisms prepared the way for a secondary infection.

*Guinea-pig* 1. The same suspension used as for Rabbit 1 ; one cubic centimetre was injected into the peritoneal cavity, and one-quarter cubic centimetre into the subcutaneous tissue of the abdomen. A few days after inoculation a very marked subcutaneous swelling extended over most of the belly. This gradually decreased in size, leaving only a small local enlargement where the needle entered. This animal was killed twenty-eight days after inoculation. Autopsy : In the subcutaneous tissue at the point of inoculation was a firm, irregular, double pea-sized mass, consisting of several translucent, gray nodules. There was a small pea-sized enlargement of an inguinal lymph node near the seat of inoculation, and an inguinal lymph node on the opposite side of the body was slightly enlarged. On the parietal peritoneum opposite the point of injection was a group of about twenty isolated, pin-head, firm, grayish foci, having upon close examination a very delicate honey-combed structure. All other organs were macroscopically normal.

### INOCULATIONS WITH PURE CULTURES.

The material used for the following inoculations was prepared by adding a small amount of the original potato culture fourteen days old to sterile water and thoroughly mixing, so that the water became slightly cloudy.

*Rabbit* 2. One cubic centimetre of the above fluid was injected into the peritoneal cavity, and one-half cubic centimetre into the right testis. The animal died four days later of peritonitis. No blastomycetes could be found in the cloudy fluid contained in the abdominal cavity, but a potato culture made from this developed three colonies of blastomycetes. The testis was greatly swollen and hemorrhagic.

*Guinea-pig* 2. One cubic centimetre was injected into the peritoneal cavity and one-half cubic centimetre into the tissue of the right mamma. Two days later there was a swelling the size of a horse-chestnut in the right inguinal region. This gradually decreased in size as time passed, and in several places small openings appeared in the skin over the nodule from which there was, especially upon pressure, a yellowish, gelatinous discharge, containing leucocytes and numerous blastomycetes. The animal was killed fifteen days after inoculation. Autopsy: Occupying the position of the right mamma was a nodule the size of a nut. It was loosely attached to the abdominal muscles by a caseous mass. (Smears from this showed no blastomycetes, but a potato culture from the same developed several colonies.) The rest of the nodule was myxomatous in character and inclosed an enlarged lymph node. The nodule was surrounded by a decidedly thick connective tissue capsule. The internal organs were normal.

The following animals were inoculated with the original potato culture one month old mixed with water as in the previous experiments:

*Rabbit* 4. Twenty minims were injected into the abdominal cavity, and five minims into the tissue of the right testicle. The animal was killed ten days after inoculation. Autopsy: A slight amount of clear, light-red serum was present in the peritoneal cavity. Cultures from this remained sterile. The right testicle had doubled in size and was immovable in the scrotum. On section there was much œdema of the scrotum, and the gland was very dark red in color. Cultures were made from this organ, resulting in numerous colonies of blastomycetes. The internal organs were normal.

*Rabbit* 3. This animal received twenty-five minims in the subcutaneous tissue of the abdomen and five minims in the right testis. It died eleven days after inoculation. Autopsy: The inguinal lymph nodes near the seat of inoculation were very much enlarged and decidedly hemorrhagic. The right testicle was much increased in size, but not as large as the testicle of Rabbit 4. There was also less œdema of the scrotum and very slight injection of the gland. Peritonitis was very marked, the peritoneal cavity being filled with a cloudy, flocculent, and slightly reddish fluid; in places there were layers of fibrin. Potato cultures from this fluid remained sterile.

*Guinea-pig* 4. This animal received twenty-five minims in the abdominal cavity and five minims in the right mamma. It died thirty-three days later. Autopsy: In the inguinal region were two distinct nodules, not attached to each other and not adherent to the abdominal muscles. The more anterior nodule was three and one-half centimetres long and two centimetres in diameter, and consisted of a reddish-yellow, translucent, jelly-like mass without macroscopic evidence of any connective tissue capsule. The other nodule was five centimetres long and from one to two centimetres in diameter, and was less jelly-like in appearance. Smears were positive, and cultures gave a profuse growth of blastomycetes. The inguinal lymph nodes on the opposite side were enlarged and showed macroscopic evidence of blastomycetic invasion. On the peritoneum toward the median line were small, isolated, translucent foci which became more numerous superiorly, soon coalescing and forming a thick, yellowish to light red, smooth, jelly-like layer upon the peritoneum, its greatest thickness being one and one-quarter centimetres. On the omentum was a yellowish-red mass of jelly seven and one-half centimetres long, and from one to four centimetres in thickness. This was more or less rough and nodulated, and in two places so deeply furrowed as to give the lesion the appearance of consisting of three distinct nodules. From this main lesion and following the line of vessels there ran out upon the omentum several strands of reddish-yellow jelly about nine centimetres long and one-half to one centimetre wide. In another portion of the omentum were isolated, translucent, jelly-like foci varying in size from a pin's head to a small pea. Cultures from the omental lesion gave an extensive growth of blastomycetes. Here and there in the mesentery were a few isolated pin-head foci, typical of blastomycetic infection. The mesenteric glands were not involved. The other organs showed no macroscopic lesions, and microscopically the kidneys were free from blastomycetes.

*Guinea-pig* 3. Inoculated with twenty-five minims in the abdominal cavity and with five minims in the right mamma. The usual subcutaneous swelling occurred in a few days, growing very large, rupturing the skin in several places, and discharging quantities of purulent, translucent, gelatinous material filled with blastomycetes. At the time of death the nodule was somewhat smaller than it had been some weeks previously. The animal died forty-eight days after inoculation. Autopsy: The subcutaneous lesions, including changes in the lymph nodes, were quite similar to those found in guinea-pig 4, as was also the condition of the omentum, though in this case it was less marked. On the peritoneum there was but one split-pea sized focus. On one fold of mesentery were about twenty pin-head foci and one nodule twice the size of a pea. The mesenteric glands were free from invasion. In this animal there were metastases in the other organs. In the spleen were five pin-head to split-pea foci. The right lobe of the liver was firmly attached to the right suprarenal and to the right kidney. The right kidney contained two small foci, the larger split-pea in size, and in the hilus was an elongated gelatinous mass. There were also numerous foci in the lungs.

HISTOLOGY. The microscopic appearance of the lesions produced in the experiment animals by inoculation of this torula is briefly as follows:*

*Subcutaneous Lesions.* The nodules formed at the seat of inoculation consisted of delicate trabeculæ of connective tissue closely woven together and forming a fine network. The spaces thus formed were filled with a homogeneous gelatinous mass in which were numerous blastomycetes of different sizes, and rarely a few cells, some probably endothelial, with vesicular nuclei, and others connective tissue cells. An occasional giant cell was seen generally toward the limiting edge of the nodule, but these and other cells were never as prominent throughout the nodule as in the original lesion in the horse. The connective tissue surrounding the nodule varied greatly in amount. Sometimes it was decidedly in evidence, as in the case of guinea-pig 2, or it was exceedingly slight in amount. The delicate fibres of connective tissue which ramify throughout the myxomatous portion of the lesion were often accompanied by small bloodvessels. Twice a *lymph node* was found within the gelatinous nodule. It was enlarged and more or less invaded with blastomycetes, which showed their characteristic envelopment interlaced with connective tissue trabeculæ. In these nodes more nuclei were found among the blastomycetes than in the subcutaneous lesions. An occasional giant cell was also present. The advancing edge showed the usual tissue reaction to a foreign body. Lymph nodes outside of the

* Tissues were hardened in 10 per cent. formalin or in Zenker's fluid; cut in paraffin and stained by Nichols'[2] method or with hæmatoxylin and eosin.

nodule on the opposite side of the body, for example, showed similar changes, all identical in character to the original lesion.

Sections of the nodule resulting from *mammary gland* inoculations were similar to those just described. Gland tissue was difficult to discover, but when found the epithelium was normal.

Sections of the *testicle* (two weeks after inoculation) showed great extravasation of serum, leucocytes, and red blood corpuscles. The glandular epithelium was almost everywhere necrotic, the lumen of the tubules filled with necrotic detritus and spermatozoa. Blastomycetes were very scarce, a single individual being found here and there throughout the organ. Only in two small areas were several, six to ten blastomycetes collected together, and in these regions the usual delicate trabeculæ of connective tissue and the gelatinous deposit about the organisms was evident. Immediately surrounding such foci were epithelioid cells and the usual accompaniments of tissue reaction.

*Peritoneum.* The lesions here, whether isolated foci or covering the membrane in a thick, uniform gelatinous layer, were identical with those heretofore described, viz., blastomycetes surrounded by a gelatinous material and bound together by a delicate network of connective tissue, such nuclei as were present being those of the connective tissue.

The lesions in the spleen, kidney, and lungs of guinea-pig 3 have not yet been studied histologically, but since they are macroscopically identical with the lesions described elsewhere and unmistakably typical of blastomycetic infection, it is fair to assume that macroscopically they will show the same type.

### CONCLUSIONS.

1. The lung lesion in the horse was caused by a torula; whether identical with Klein's, Plimmer's, or one of Sanfelice's is not yet determined.

2. Inoculations of rabbits and guinea-pigs with material from the original lesion and with pure cultures of the torula obtained from it, reproduced similar lesions in these animals, and if time permitted, metastases.

3. This torula produces a purely inflammatory reaction in tissues. It does not cause a proliferation of epithelial cells; it may cause a necrosis of them or it may leave them uninjured.

4. The results of my experiments are identical with those of Nichols, though I seem to have been working with a more virulent organism.

REFERENCES.

1 Ricketts. Oidiomycosis (Blastomycosis) of the Skin and its Fungi. Journ. of Medical Research, vol. vi., No. 3. Whole No. 64.

2 Nichols. Relation of Blastomycetes to Cancer. Ibid., vol. vii., No. 3. Whole No. 67.

3 Fermi and Aruch. Ueber eine neue pathogene Hefeart und die natur des sogenannten Cryptococcus farcinimosus Rivoltæ. Centr. f. Bakt. u. Par., 1895, B. xvii., p. 593.

4 Tokishige. Ueber Pathogene Blastomyceten. Ibid., 1896, B. xix., p. 105.

5 Weis. Four Pathogenic Torulæ. Journ. of Medical Research, vol. vii., No. 3. Whole No. 67.

6 Klöcker. Die Gärungsorganismen in der Teorie und Praxis der Alkoholgärungsgewerbe. Stuttgart, 1900.

## Figures and Tables

**Figure f1:**